# Efficacy of early goal-directed therapy using FloTrac/EV1000 to improve postoperative outcomes in patients undergoing off-pump coronary artery bypass surgery: a randomized controlled trial

**DOI:** 10.1186/s13019-022-01933-4

**Published:** 2022-08-21

**Authors:** Sirirat Tribuddharat, Thepakorn Sathitkarnmanee, Kriangsak Ngamsaengsirisup, Sanpicha Sornpirom

**Affiliations:** 1 Department of Anesthesiology, Faculty of Medicine, Khon Kaen University, 123 Mitrapap Road, Ampur Muang, Khon Kaen, 40002 Thailand; 2 Cardiothoracic Intensive Care Unit, Faculty of Medicine, Khon Kaen University, 123 Mitrapap Road, Ampur Muang, Khon Kaen, 40002 Thailand

**Keywords:** Early goal-directed therapy, Off-pump coronary artery bypass graft, Length of stay, Intensive care unit, Hospital

## Abstract

**Background:**

Early goal-directed therapy (EGDT) using FloTrac reduced length of stay (LOS) in intensive care (ICU) and hospital among patients undergoing coronary artery bypass graft (CABG) with a cardiopulmonary bypass. However, this platform in off-pump CABG (OPCAB) has received scant attention, so we evaluated the efficacy of EGDT using FloTrac/EV1000 as a modality for improving postoperative outcomes in patients undergoing OPCAB.

**Methods:**

Forty patients undergoing OPCAB were randomized to the EV1000 or Control group. The Control group received fluid, inotropic, or vasoactive drugs (at the discretion of the attending anesthesiologist) to maintain a mean arterial pressure 65–90 mmHg; central venous pressure 8–12 mmHg; urine output ≥ 0.5 mL kg^−1^ h^−1^; SpO_2_ > 95%; and hematocrit ≥ 30%. The EV1000 group achieved identical targets using information from the FloTrac/EV1000. The goals included stroke volume variation < 13%; cardiac index (CI) of 2.2–4.0 L min^−1^ m^−2^; and systemic vascular resistance index of 1500–2500 dynes s^−1^ cm^−5^ m^−2^.

**Results:**

The EV1000 group had a shorter LOS in ICU (mean difference − 1.3 d, 95% CI − 1.8 to − 0.8; *P* < 0.001). The ventilator time for both groups was comparable (*P* = 0.316), but the hospital stay for the EV1000 group was shorter (mean difference − 1.4 d, 95% CI − 2.1 to − 0.6; *P* < 0.001).

**Conclusions:**

EGDT using FloTrac/EV1000 compared to conventional protocol reduces LOS in ICU and hospital among patients undergoing OPCAB.

*Trial registration* This study was retrospectively registered at www.ClinicalTrials.gov (NCT04292951) on 3 March 2020.

## Background

Coronary artery bypass graft (CABG)—including cardiopulmonary bypass (CPB) or on-pump CABG (ONCAB)—can induce many deleterious effects mediated by a systemic inflammatory response as a result of contact of blood elements with surfaces of the CPB system. Adverse effects could include postoperative myocardial, renal, and neurological dysfunction, coagulopathies, respiratory failure, and multiple organ dysfunction [1]. To avoid such harmful effects, CABG has been performed on the beating heart without CPB or off-pump CABG (OPCAB). A large, randomized, controlled trial (RCT) —the CORONARY—revealed that OPCAB, in contrast to ONCAB, reduced the rate of (a) transfusion, (b) reoperation for bleeding, (c) acute renal injury, and (d) respiratory complications albeit increased the risk of early revascularization [2]. However, no differences were reported in death, non-fatal stroke, or non-fatal myocardial infarction (MI) at 30 days between OPCAB and ONCAB in two large RCTs—the CORONARY and ROOBY [2–4]. Nevertheless, some patients with compromised left ventricular function may benefit from OPCAB by avoiding CPB. In parallel with the development of epicardial stabilizing devices and the improvement of the surgeon learning curve, there has been an upsurge in the number of OPCAB procedures being performed [5]. During OPCAB, manipulation of the beating heart caused fluctuations in patient hemodynamics such as decreased systemic arterial pressure (SAP) and cardiac index (CI), and increased mean pulmonary arterial pressure (PAP) [6]. These changes compromise the oxygen supply to the vulnerable myocardium, leading to an increased (a) requirement for inotropic and vasoactive drugs, (b) duration of mechanical ventilation, and (c) length of stay (LOS) both in the intensive care unit (ICU) and hospital [5].

Early goal-directed therapy (EGDT) is a method for hemodynamic optimization. EGDT is achieved by administering fluid as well as inotropic and vasoactive drugs to maintain preload, contractility, and afterload within an optimal range [7]. EGDT improves perioperative outcomes by decreasing the LOS in ICU and hospital among patients undergoing high-risk surgeries [8, 9]. The techniques used to determine the EGDT goals include PiCCO plus, FloTrac/EV1000, esophageal Doppler, and thermodilution pulmonary artery catheter. FloTrac/EV1000 is a minimally invasive monitor with no need for calibration. It is connected with a standard radial artery catheter and a central venous pressure (CVP) catheter. Stroke volume is computed from the arterial waveform using a proprietary algorithm for pulse contour analysis. It can also provide continuous real-time values (updated every 20 s) of stroke volume variation (SVV), stroke volume index (SVI), CI, and systemic vascular resistance index (SVRI). The SVV was initially validated for predicting fluid responsiveness in closed-chest patients and not recommended for open-chest. Some studies have lately validated the use of SVV in open-chest contexts by showing that SVV and pulse pressure variation (PPV) could be used to predict fluid responsiveness in patients under open-chest or open-pericardial conditions [10, 11]. Several studies have shown that EGDT using FloTrac reduced LOS in ICU and hospital in patients undergoing ONCAB [7, 12–15], while few studies have assessed the benefits of EGDT in OPCAB [5, 16]. Our objective was to evaluate the efficacy of applying EGDT using the FloTrac/EV1000 platform to improve postoperative outcomes in patients undergoing OPCAB. Our working hypothesis was that the system would decrease LOS in the ICU and hospital.

## Materials and Methods

The Khon Kaen University Ethics Committee in Human Research approved the study (HE611321) on 11 September 2019. The study was registered at www.ClinicalTrials.gov (NCT04292951) on 3 March 2020. The first case of the study was recruited on 1 January 2020, the other were recruited after 3 March 2020. We failed to register before participant recruitment due to miscommunication in the team. All methods were performed in accordance with the Declaration of Helsinki and the ICH GCP, and all participants gave written informed consent before being recruited. Study reporting followed the Consolidated Standards of Reporting Trials (CONSORT) guidelines.

This study was a prospective, randomized, double-blind (patient- and assessor-blinded) controlled trial. The sample size calculation was based on an ICU LOS after OPCAB of 4.20 ± 0.82 d, as reported in a previous study [5]. Twenty patients per group were needed to detect a 35% decrease in ICU LOS with an α-value of 0.05, a power (1-ß) of 0.80, and a 20% dropout. Block-of-4 randomization with a 1:1 allocation ratio was performed using a computer-generated list kept in sealed opaque envelopes. The included patients were 1) male or female; 2) 18 or over; 3) undergoing OPCAB under general anesthesia at Srinagarind Hospital or Queen Sirikit Heart Center of the Northeast, Khon Kaen University, Khon Kaen, Thailand; and, 4) between II and IV on the American Society of Anesthesiologists (ASA) classification. Patients were excluded if (1) urgent or repeat surgery was required; (2) a contraindication existed vis-à-vis central venous cannulation; (3) an intra-aortic balloon pump was needed; (4) ventricular arrhythmia was present; or, (5) they were unable or unwilling to cooperate. The surgeons and anesthesiologists had experience with at least 120 cases of OPCAB surgery.

Patients were randomized to the EV1000 or Control group. Patients received standard cardiac anesthesia care as per standard protocol. Operative monitoring included electrocardiogram, pulse oximeter, non-invasive blood pressure, temperature, capnography, anesthetic gas analyzer, and urine output. In the Control group, the radial artery was cannulated and connected to a pressure transducer to measure invasive blood pressure. In the EV1000 group, the radial artery was cannulated and connected to a FloTrac transducer (Edwards Lifesciences, Irvine, CA, USA) connected to an EV1000 monitor (Edwards Lifesciences, Irvine, CA, USA) to measure invasive blood pressure, SVV, CI, and SVI. In the Control group, the internal jugular vein was cannulated and connected to another pressure transducer to measure CVP. In the EV1000 group, the internal jugular vein was cannulated and connected to a pressure transducer which was connected to the EV1000 monitor to measure SVRI.

Irrespective of group, induction was achieved by a premedication of fentanyl 2–3 µg·kg^−1^ and midazolam 1 mg, propofol 2–3 mg·kg^−1^ or etomidate 0.3 mg·kg^−1^. Cis-atracurium 0.2 mg·kg^−1^ was used to facilitate endotracheal intubation. Anesthesia was maintained with 50% oxygen in the air and 1–2% sevoflurane or 3–6% desflurane—adjusted to 1 minimum alveolar concentration (MAC) on the monitor to maintain the depth of anesthesia. Heparin 1.5–2 mg·kg^−1^ was administered via the CVP catheter for an activated clotting time (ACT) of 300–350 s with additional doses to maintain ACT of 250–300 s. After the operation, protamine 0.3–0.5 mg per 1 mg of the initial dose of heparin was given stepwise to reverse the effect of heparin. Patients were moved to ICU, where they were mechanically ventilated and given standard care.

Ventilator weaning and extubation were performed according to (1) good consciousness and motor power; (2) stabilized cardiovascular status; (3) PaO_2_/FiO_2_ ratio ≥ 250 mmHg; and, 4) respiratory rate 10–25 times·min^−1^. The ICU discharge criteria included (1) satisfactory consciousness and neurological signs; (2) steady cardiovascular status without any need for inotropic or vasopressor drugs or ICU monitoring; and, (3) a satisfactory respiratory status not requiring greater than 60% oxygen supplementation. The benchmarks for hospital discharge included (1) stable cardiovascular and respiratory status; (2) no drain or catheter needed; (3) normal ambulation; (4) no infection or serious complications; (5) no wound stitches; and, (6) normal diet.

Each patient's heart kept beating throughout the surgery. The patients were put in the Trendelenburg position during coronary anastomosis.

In the intraoperative period, the Control group received fluid, inotropic, or vasoactive drugs as per the attending anesthesiologist. The goals were: mean arterial pressure (MAP) 65–90 mmHg; CVP 8–12 mmHg; urine output ≥ 0.5 mL·kg^−1^·h^−1^; SpO_2_ > 95%; and hematocrit ≥ 30%. Arterial blood gas (ABG) and electrolytes were monitored hourly and corrected as required. The goals for patients in the EV1000 group were the same—i.e., MAP 65–90 mmHg; urine output ≥ 0.5 mL·kg^−1^·h^−1^; SpO_2_ > 95%; and hematocrit ≥ 30%—but achieved using the EGDT protocol with data from the FloTrac/EV1000. Patients first received fluid to maintain SVV < 13%, followed by inotropic drugs to maintain a CI of 2.2–4.0 L·min^−1^·m^−2^, followed by vasoactive drugs to maintain an SVRI of 1500–2500 dynes s^−1^·cm^−5^·m^−2^. ABG and electrolytes were likewise monitored and corrected.

The recorded parameters included: volume of fluid, amount of inotropic and vasoactive drugs used during the intraoperative period, transfer to ICU, and in ICU. Also recorded were the duration of mechanical ventilation in the ICU, LOS in ICU and hospital, and complications.

### Statistical analysis

The Shapiro–Wilk test was used to determine whether the continuous data were normally distributed. Data with a normal distribution were presented as means ± standard deviations (SD), while non-normal distributed data were presented as medians with interquartile ranges. Categorical data were presented as numbers (%). As appropriate, the differences between both groups were analyzed using the unpaired Student’s t-test, Mann–Whitney U test, χ^2^ test, or Fisher exact test. *P* < 0.05 was considered statistically significant. Statistical analyses were performed using SPSS 16.0 for Windows (SPSS, Chicago, IL, USA).

## Results

**Fig. 1:**
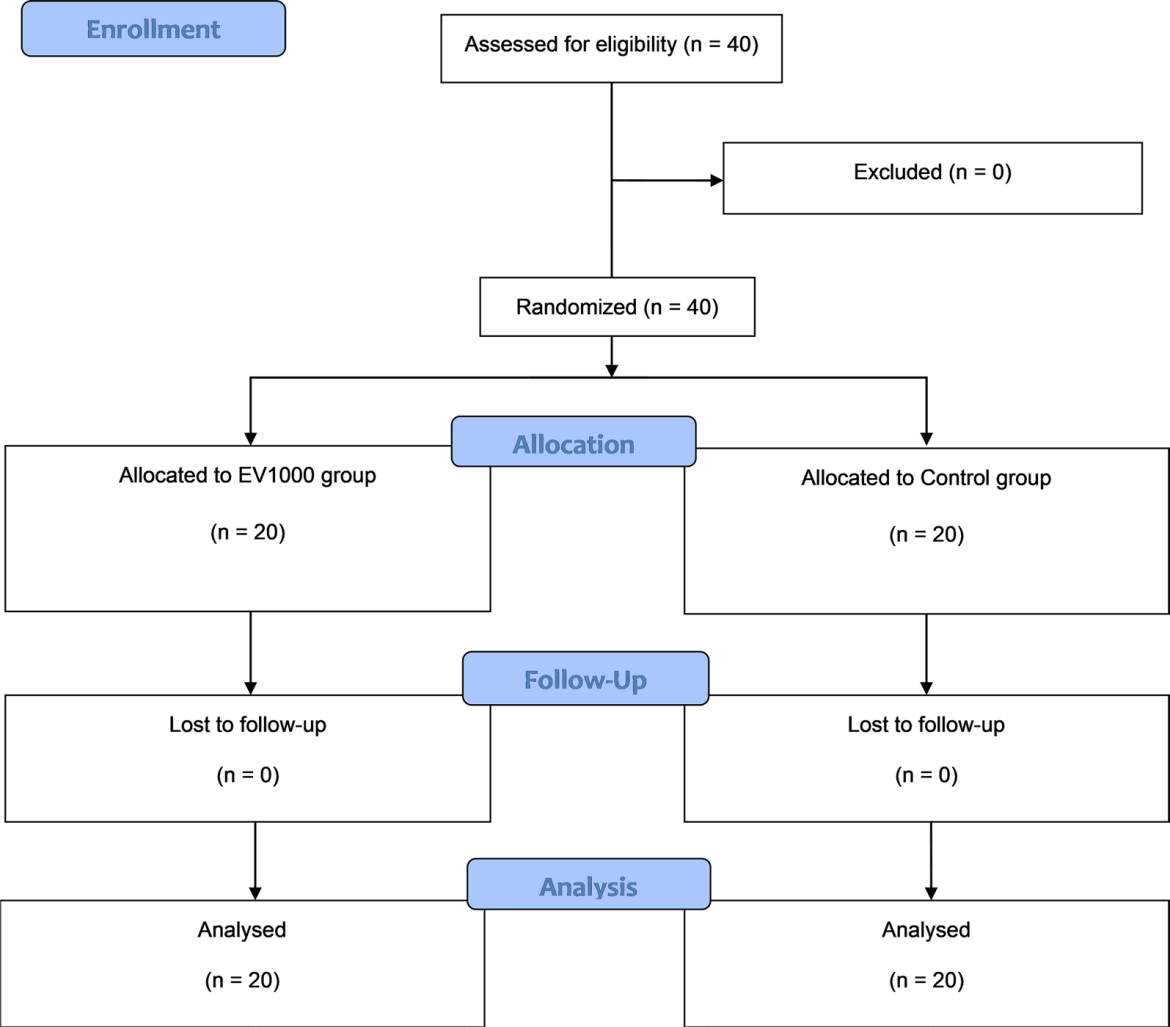
COSORT diagram of the study

Forty patients with 20 in each group were included and analyzed during 1 January 2020 and 1 July 2020 as shown in the CONSORT flow diagram (Fig. 1). The patient characteristics and clinical data were similar between both groups. However, the EV1000 group received more crystalloid intake and had more urine output than the Control group (Table 1).

**Table 1:** Characteristics and clinical data of patients (n = 40)

Variable	EV1000 (n = 20)	Control (n = 20)	*P*-value
Age (y)	68.1 ± 5.7	65.1 ± 9.3	0.226
Sex: male	14 (70)	16 (80)	0.328
Height (cm)	163.2 ± 7.1	161.1 ± 7.3	0.328
Weight (kg)	64.3 ± 12.2	70.0 ± 11.8	0.140
Number of vessel anastomosis			0.101
2	2 (10)	1 (5)	
3	11 (55)	7 (35)	
4	7 (35)	7 (35)	
5	0 (0)	5 (25)	
Functional class			0.128
2	8 (40)	14 (70)	
3	11 (55)	6 (30)	
4	1 (5)	0 (0)	
ASA classification			0.116
2	2 (10)	7 (35)	
3	17 (85)	13 (65)	
4	1 (5)	0 (0)	
Ejection fraction (%)	59.3 ± 6.6	63.3 ± 13.8	0.253
Anesthesia time (min)	309.8 ± 72.6	301.5 ± 42.7	0.664
Crystalloid intake (mL/h)	572.0 ± 72.3	427.3 ± 232.4	0.014
Blood loss (mL)	680.0 ± 392.2	665.0 ± 253.4	0.887
Urine output (mL/h)	104.2 ± 23.3	65.0 ± 52.0	0.005

Data are expressed as mean ± SD or number (%)

*ASA* American Society of Anesthesiologists; *CKD* chronic kidney disease; *INR* international normalized ratio; *CPB* cardiopulmonary bypass

The EV1000 group had a shorter LOS in the ICU (mean difference − 1.3 d, 95% CI − 1.8 to − 0.8; *P* < 0.001). The ventilator time for both groups was similar (*P* = 0.316). The hospital stay for the EV1000 group was shorter (mean difference − 1.4 d, 95% CI − 2.1 to − 0.6; *P* < 0.001) (Table 2). The EV1000 group required more number of inotropic and vasoactive drugs during the intraoperative period (*P* = 0.001) but less during the immediate postoperative period (*P* < 0.001) and in the ICU (*P* = 0.005) (Table 3). The Control group had more postoperative respiratory and cardiovascular complications, albeit not statistically significant (Table 4).

**Table 2:** Postoperative outcomes

	EV1000 (n = 20)	Control (n = 20)	Mean difference	95% CI	*P*-value
Ventilator time (h)	15.2 ± 3.8	16.9 ± 6.4	− 0.2	− 6.0 to 5.6	0.316
ICU stay (d)	1.7 ± 0.1	3.0 ± 1.0	− 1.3	− 1.8 to − 0.8	< 0.001
Hospital stay (d)	8.8 ± 1.2	10.2 ± 1.1	− 1.4	− 2.1 to − 0.6	< 0.001

Values are presented as mean ± SD

*ICU* intensive care unit

**Table 3:** Number of inotropic or vasoactive drugs required at different stages for each group (n = 40)

	EV1000 (n = 20)	Control (n = 20)	*P*-value
Number of drug requirement during intraoperative period			0.001
0	0 (0)	3 (15)	
1	4 (20)	13 (65)	
2	13 (65)	4 (20)	
3	3 (15)	0 (0)	
Number of drug requirement during immediate postoperative period			< 0.001
0	18 (90)	2 (10)	
1	1 (5)	13 (65)	
2	1 (5)	5 (25)	
Number of drug requirement in ICU			0.005
0	7 (35)	0 (0)	
1	9 (45)	5 (25)	
2	2 (10)	9 (45)	
3	2 (10)	5 (25)	
4	0 (0)	1 (5)	

Values are expressed as number (%)

*ICU* intensive care unit

**Table 4:** Postoperative complications for each group

	EV1000 (n = 20)	Control (n = 20)	*P*-value
*Postoperative complication*			
Pleural effusion	2 (10)	3 (15)	1.000
AF with RVR	1 (5)	4 (20)	0.342
VT/VF	0 (0)	2 (10)	0.487
Lung congestion	0 (0)	1 (5)	1.000
ARDS	0 (0)	2 (10)	0.487
On IABP	0 (0)	2 (10)	0.487

Values are presented as number (%)

*AF with RVR* atrial fibrillation with rapid ventricular response rate; *VT/VF* ventricular tachycardia/ventricular fibrillation; *ARDS* acute respiratory distress syndrome; *IABP* intra-aortic balloon pump

## Discussion

Our study revealed that hemodynamic optimization using EGDT via FloTrac/EV1000 platform, compared with standard care, reduced the LOS in ICU and hospital among patients undergoing OPCAB. The results agree with other studies. For example, Smetkin et al. used continuous monitoring of S_CV_O_2_ as guidance for EGDT in patients undergoing OPCAB, resulting in decreased LOS in ICU and hospital by 15% and 25%, respectively [16]. Cao et al*.* observed that applying EGDT using SVV and CI as targets for hemodynamic optimization among patients undergoing OPCAB reduced hospital stay by 19.4% (95% CI 7.3 to 1.5%) [17]. Kapoor et al. assessed the efficacy of EGDT using FloTrac/EV1000 in patients undergoing OPCAB. They concluded that EGDT using FloTrac/EV1000 reduced LOS in ICU (4.20 ± 0.82 vs. 2.53 ± 0.56 days, *P* < 0.001) and hospital (7.42 ± 1.48 vs. 5.61 ± 1.11 days, *P* < 0.001) [5]. Slagt, however, questioned the validity of this study regarding the lack of definite endpoint criteria [18]. Our study defined the exact criteria for each endpoint and continued to have similar results rebutting Slagt’s concerns.

ICU costs account for most of the expenditure related to cardiac surgery. For example, the average daily ICU fee for cardiac surgery for moderate-risk patients—as reported by a tertiary hospital in Brazil—was US$ 1225.77 [19]. Thus, an ICU stay shortened by − 1.3 d represents a significant cost reduction (US$ 1613.93) and improvement in ICU bed management so that other patients can be admitted and treated.

Ischemic heart disease compromises the oxygen demand–supply balance of the myocardium. During OPCAB, manipulating the beating heart can result in variations in patient hemodynamics, such as decreased systolic blood pressure (SBP) and CI, and increased mean PAP [6]. These variations can further jeopardize oxygen supply to the myocardium, trending toward a greater need for inotropic and vasoactive drugs during the immediate postoperative period and ICU, resulting in greater LOS in the ICU. Early detection and prompt treatment of low cardiac output would improve outcomes [7]. Most anesthesiologists aim for a MAP between 90 and 105 mmHg to avoid low cardiac output [7]. MAP does not necessarily reflect cardiac output because it is a function of cardiac output and systemic vascular resistance (SVR). At the same time, cardiac output depends on preload and contractility. Thus, three variables influence MAP—viz. preload, contractility, and afterload or SVR. A normal MAP can occur when cardiac output is low with a high SVR, resulting in tissue hypoperfusion. Treatment of intraoperative hypotension without knowledge of these three variables could lead to mismanagement, increasing pharmacologic requirements, complications, and the LOS in ICU and hospital. These three variables are continuously monitored in EGDT and promptly managed to optimize blood pressure and flow for optimal outcomes.

Albeit not statistically significant, the Control group has five patients (25%) with five vessel anastomosis compared with none in the EV1000 group indicating a greater need for heart mobilization during surgery, and commonly requiring more fluid and drugs. The EV100 group still receive more fluid and drugs during intraoperative period because of the fine-tuning protocol based on the information derived from the FloTrac/EV1000 platform.

EGDT applied intraoperatively improves postoperative outcomes in non-cardiac major surgery [8, 9] and cardiac surgery [15, 20, 21]. Several means are available to assess EGDT goals (viz., PICCO Plus, FloTrac/EV1000, esophageal Doppler, and thermodilution pulmonary artery catheter) [20]. A fourth-generation FloTrac with EV1000 was used to support the goals for EGDT as the platform is minimally invasive, easy to use, and provides real-time data. Although a limitation of SVV is that it is not recommended for open thorax, its efficacy has been confirmed for assessing fluid responsiveness and improving postoperative outcomes in open-chest surgery [10, 11, 21].

As with previous studies, to achieve a target MAP, the EGDT group required more fluid and drugs during the intraoperative period to optimize SVV, CI, and SVRI based on information from the FloTrac/EV1000 [15, 16]. These achievements resulted in better myocardium perfusion leading to less drug requirement during the immediate postoperative period and in the ICU, leading to a shorter ICU and hospital LOS. Taken together, the EGDT group developed fewer postoperative complications than the Control group, as Tribuddharat et al. reported [15]. Although the differences in postoperative complications did not achieve statistical significance, they may reflect clinical significance. The assumption is that the fewer complications in the EGDT group compared to the Control group was the result of more optimized tissue perfusion during the intraoperative period.

Although uncertainty remains regarding cardiac output measurement in cardiac surgery using the FloTrac/Vigileo system versus the thermodilution technique [22–24], the current study and others demonstrate that using the FloTrac/EV1000 system in OPCAB surgery can improve postoperative clinical outcomes.

The study did not employ a bi-spectral index (BIS) for regulating the depth of anesthesia; however, the minimum alveolar concentration (MAC) value was used to monitor and control the depth of anesthesia. The end-tidal concentration of sevoflurane or desflurane was adjusted to achieve 1 MAC for a given age (MAC_age_) [25]. The effect of fentanyl ~ 0.5 MAC and cisatracurium ~ 0.5 MAC [26, 27], resulted in an optimal total depth of anesthesia of about 2 MAC (~ MAC required to block adrenergic response in 99% of patients (MAC-BAR_99_)).

The study had some limitations. First, the attending anesthesiologists were not blinded; notwithstanding, the attending physicians and nurses in the ICU were unaware of the intraoperative hemodynamic management protocol. Second, the sample size was small and confined to a single tertiary care center, so a larger, multi-center, follow-on study is needed. The strength of the study is that both the Control and EV1000 groups were managed intraoperatively by highly experienced anesthesiologists so as to achieve the same clinical goals despite the different monitoring platforms. The high cost of the FloTrac/EV1000 platform may be offset by reductions in ICU and hospital LOS. A cost-effectiveness study regarding this aspect is suggested.

## Conclusions

Compared with conventional care, hemodynamic optimization with EGDT protocol using FloTrac/EV1000 in patients undergoing OPCAB surgery reduces LOS in both ICU and hospital.

## Data Availability

The data used to support the findings of this study are available from the corresponding author upon request.

## Abbreviations

CABG: Coronary artery bypass graft
CPB: Cardiopulmonary bypass
ONCAB: On-pump CABG
OPCAB: Off-pump CABG
RCT: Randomized controlled trial
SAP: Systemic arterial pressure
CI: Cardiac index
PAP: Pulmonary arterial pressure
LOS: Length of stay
ICU: Intensive care unit
EGDT: Early goal-directed therapy
CVP: Central venous pressure
SVV: Stroke volume variation
SVI: Stroke volume index
SVRI: Systemic vascular resistance index
PPV: Pulse pressure variation
CONSORT: Consolidated Standards of Reporting Trials
MAP: Mean arterial pressure
ABG: Arterial blood gas
ARDS: Acute respiratory distress syndrome
PONV: Postoperative nausea and/or vomiting
SBP: Systolic blood pressure
SVR: Systemic vascular resistance
MAC: Minimum alveolar concentration
MAC_age_: MAC for a given age
MAC-BAR_99_: MAC required to block adrenergic response in 99% of patients
